# A new species of *Peckoltia* from the Upper Orinoco (Siluriformes, Loricariidae)

**DOI:** 10.3897/zookeys.569.6630

**Published:** 2016-02-26

**Authors:** Jonathan W. Armbruster, Nathan K. Lujan

**Affiliations:** 1 Department of Biological Sciences, 101 Life Sciences Building, Auburn University, AL 36849, USA; 2 Department of Biological Science, University of Toronto Scarborough, Toronto, ON M1C1A4, Canada

**Keywords:** Ancistrini, Hypostominae, Molecular Phylogeny, Morphology, Peckoltia, Systematics, Taxonomy

## Abstract

A new species of the suckermouth armored catfish genus *Peckoltia* is described from the lower Ventuari River, a tributary of the upper Orinoco River in Amazonas State, Venezuela. Specimens of this species were formerly included in the wide-ranging Amazonian species *Peckoltia vittata*, but a recent molecular phylogeny found Orinoco individuals to be distantly related to Amazon Basin individuals spanning the range of *Peckoltia vittata* syntypes. Detailed morphological examination confirmed distinctiveness of Orinoco specimens, and found them to be diagnosable from true *Peckoltia vittata* by having generally greater than 25 teeth (vs. less), spots on the nape (vs. nape lacking spots), the upper lip with two to three black bar-shaped markings in a line like a moustache (vs. lips generally with a hyaline wash), and by the snout having a medial black line disconnected from the moustache markings (vs. medial snout stripe connected to a bar just above the lip). *Peckoltia wernekei* displays remarkable genetic similarity to its sister species, *Peckoltia lujani*, but differs morphologically by having dentary tooth rows meet at an angle less than 90° (vs. >90°), by having large faint blotches on the abdomen (vs. abdomen with no blotches), by a smaller internares width (21.2–26.6% vs. 28.5–46.5% of interorbital width), and a larger dorsal spine (148.1–178.6% vs. 80.1–134.5% of abdominal length).

## Introduction

*Peckoltia* Miranda Ribeiro, 1912 is a genus of suckermouth armored catfishes (Loricariidae) with 18 currently described species (Armbruster et al. 2015). Armbruster (2004; 2008) had restricted *Peckoltia* to those species of Ancistrini that had dentary tooth rows meeting at an angle of 90° or less and that lacked the synapomorphies of similar genera like *Hypancistrus* and *Panaqolus* (the latter then the *Panaque dentex* group); however, this arbitrary definition was not supported by the molecular analysis of Lujan et al. (2015), so Armbruster et al. (2015) recognized an expanded *Peckoltia* that currently lacks a morphological diagnosis, but is strongly monophyletic based on molecular evidence (Lujan et al. 2015).

**Figure 1.**
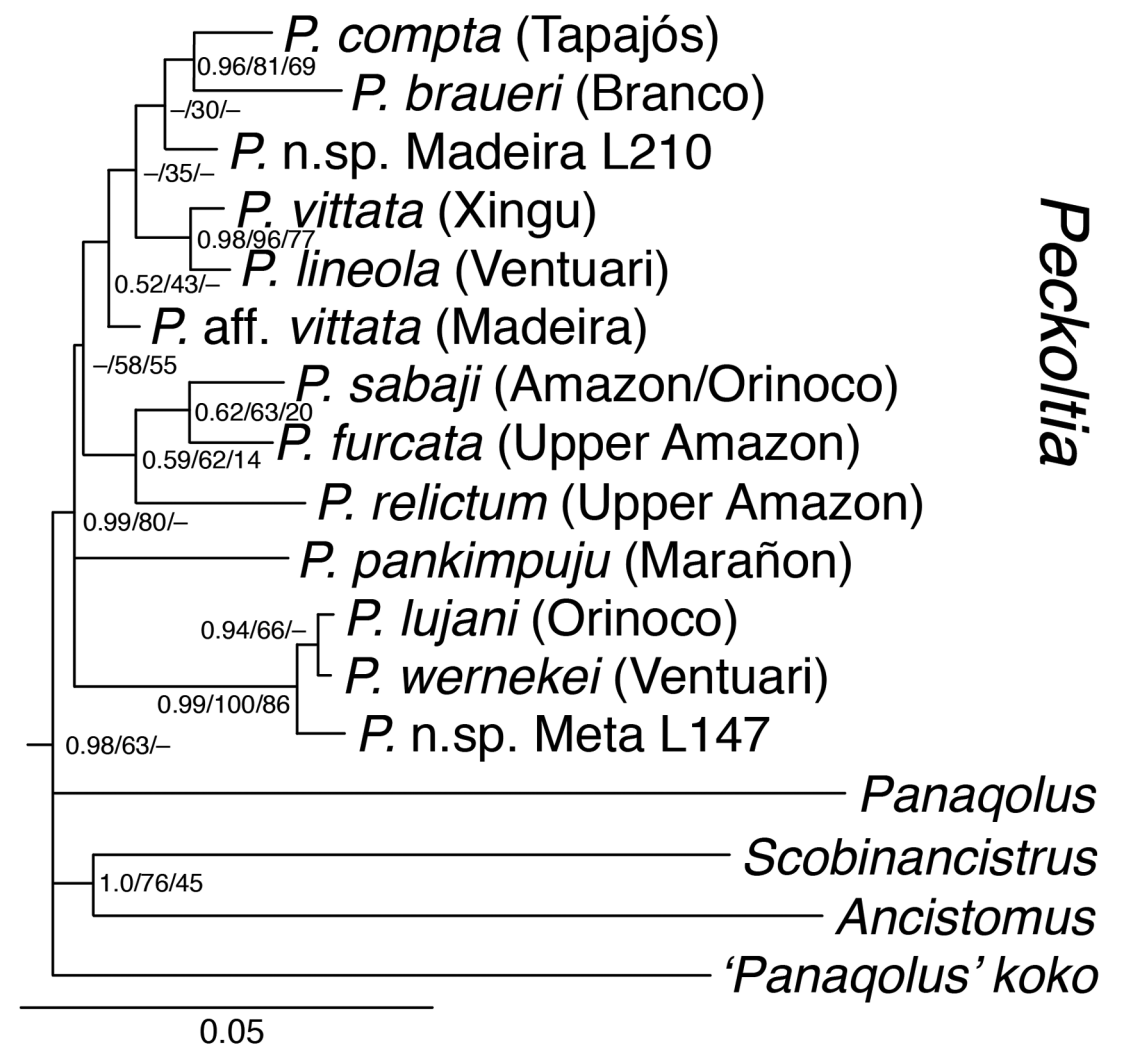
Phylogenetic relationships within the *Peckoltia* Clade (sensu Lujan et al. 2015), from Lujan et al. (in review). Results based on analysis of a 4293 base pair alignment consisting of two mitochondrial (16S, Cyt b) and three nuclear loci (RAG1, RAG2, MyH6). Node support values given in order as Bayesian posterior probability, maximum likelihood bootstrap and maximum parsimony bootstrap.

Armbruster (2008) had recognized a wide-ranging *Peckoltia vittata* (Steindachner, 1881) that included specimens from the Maranhão to the Madeira and upper Orinoco, but suggested that this putative range of *Peckoltia vittata* included multiple species. The syntype series of *Peckoltia vittata* itself contains specimens spanning more than 600 km of the main channel of the Amazon River, from the Xingu River to the Madeira River, making it difficult to know where one might reliably find the true *Peckoltia vittata*. Specimens from the Xingu, Madeira and Orinoco that were morphologically consistent with *Peckoltia vittata* were found to be polyphyletic in Lujan et al. (2015) and Lujan et al. (in review, Fig. 1). In addition, *Peckoltia greedoi* was recognized as distinct from *Peckoltia vittata* by Armbruster et al. (2015; specimens of *Peckoltia greedoi* were originally included among the specimens of *Peckoltia vittata* examined by Armbruster 2008).

Lujan et al. (2015) found the putative specimens of *Peckoltia vittata* from the Orinoco to be strongly supported as sister to another Orinoco species, *Peckoltia lujani* Armbruster, Werneke and Tan, and part of a clade with another undescribed species known as *Peckoltia* sp. n. Meta L147. In this study, we re-examine specimens identified as *Peckoltia vittata* from the upper Orinoco and describe them as a new species clearly distinguishable from Amazon Basin *Peckoltia vittata*. In addition, we provide a key to all of the current species of *Peckoltia*.

## Methods

Methods follow Armbruster (2003) with the addition of counts of mid-dorsal and mid-ventral plates (the number of plates in these series from the head to caudal fin and excluding the last, triangular plate, which is beyond the hypural). Institutional abbreviations are as in Sabaj Pérez (2014). Names of skeletal characteristics are as in Schaefer (1987) and Geerinckx et al. (2007) and of plate rows as in Schaefer (1997). Full morphometric dataset is presented in Suppl. material 1, locality information for species described below is presented in Suppl. material 2.

## Taxonomy

### Peckoltia wernekei

Taxon classificationAnimaliaSiluriformesLoricariidae

Armbruster & Lujan sp. n.

http://zoobank.org/3488FBE3-34F0-4F5B-94D2-60BFA849D945

Figs 3 , 4 and 5b , Table 1

Peckoltia aff. vittata (Orinoco) Lujan et al., 2015 [molecular phylogeny]

#### Type locality.

Ventuari River drainage, Amazonas State, Venezuela, South America

#### Holotype.

AUM 54314, 104.6 mm SL, VENEZUELA, Amazonas State, Ventuari River drainage, Marujeta Creek, 159 km E of San Fernando de Atabapo, 04.2948°, -066.2889°, N.K. Lujan, M. Sabaj Pérez, D.C. Werneke, T. Carvalho, V. Meza-Vargas, 02 April 2010.

#### Paratypes.

All specimens Venezuela, Amazonas State, Ventuari River drainage: AUM 39248, 1, 63.2 mm SL, Ventuari River at beach at village of Moriche, 116 km NE of Macuruco, 169 km NE of San Fernando de Atabapo, 04.7503°, -066.3549°, D.C. Werneke, N.K. Lujan, M.H. Sabaj, L.S. de Souza, 7 April 2004. AUM 39313, 13, 2 cs, 56.8–87.3 mm SL, Manapiare River, 14.5 km NW of San Juan de Manapiare, 05.4286°, -066.1362°, N.K. Lujan, M.H. Sabaj, L.S. de Souza, D.C. Werneke, 12 April 2004. AUM 39839, 1, 31.5 mm SL, Manapiare River, 10 km NW of San Juan de Manapiare, 05.3868°, -066.1159°, N.K. Lujan, L.S. de Souza, D.C. Werneke, M.H. Sabaj, 14 April 2004. MCNG 56680, 13, 52.6–80.2 mm SL, same data as AUM 39313.

#### Diagnosis.

*Peckoltia wernekei* can be separated from all other *Peckoltia* by having a broken black line of pigment on the upper jaw (vs. solid line of pigment along snout edge or snout uniformly colored or mottled). *Peckoltia wernekei* can be further separated from *Peckoltia vittata* by generally having 25 or more teeth in at least one dentary or one premaxilla (vs. generally 24 or fewer; one specimen of *Peckoltia wernekei* had both upper and lower jaws with <25 teeth/ramus), by having a largely naked abdomen (abdomen with a few plates below pectoral girdle, between pelvic fins and along sides of abdomen; vs. most of ventral surface from the throat to the anus with small plates), and by having large, faint blotches on the abdomen (vs. abdomen uniform). *Peckoltia wernekei* can be further separated from upper Orinoco congeners as follows: from *Peckoltia brevis* and *Peckoltia lineola* by lacking short lines and spots on the head (vs. lines and spots present), from *Peckoltia brevis*, *Peckoltia caenosa* and *Peckoltia lineola* by having a largely naked abdomen (vs. abdomen fully plated), and by generally having 25 or more teeth per jaw ramus (vs. 22 or fewer in *Peckoltia brevis*, 21 or fewer in *Peckoltia caenosa*, and 19 or fewer in *Peckoltia lineola*); from *Peckoltia lujani* by having the dentaries meet at an angle less than 90° (vs. >90°), by having large, faint blotches on the abdomen (vs. abdomen with no blotches), by a smaller internares width to interorbital width ratio (21.2–26.6% vs. 28.5–46.5%), and a larger dorsal spine to abdominal length ratio (148.1–178.6% vs. 80.1–134.5%); and from *Peckoltia sabaji* by having bands in the dorsal and caudal fins (vs. spots) and prominent dorsal saddles on the body (vs. large spots).

#### Description.

Morphometrics in Table 1. Counts and measurements based on 23 specimens. Small to medium-sized loricariids, largest specimen examined 104.6 mm SL. Body stout, but slightly narrower than *Peckoltia vittata*. Head gently sloped to supraoccipital. Supraoccipital with tall, rounded crest. Supraoccipital crest raised slightly above nuchal region. Nuchal region rises slightly to nuchal plate. Dorsal slope decreasing in straight line to insertion of dorsal procurrent caudal-fin rays then ascending to caudal fin. Body depth greatest at anteriormost insertion of dorsal fin. Ventral profile flat to caudal fin. Caudal peduncle trapezoidal in cross section with dorsal surface flattened. Body widest at insertion of pectoral fins, narrowest at insertion of caudal fin. Snout rounded.

**Table 1.** Selected morphometrics of *Peckoltia wernekei*. Numbers in parentheses refer to landmark numbers in Armbruster (2003).

	Holotype	N	Mean	SD	Min	Max
SL, mm (1–20)	104.6	23	70.2		52.6	104.6
%SL						
Predorsal Length (1–10)	38.8	23	42.2	1.5	38.8	45.2
Head Length (1–7)	36.5	23	35.5	1.2	32.5	37.5
Head–dorsal Length (7–10)	6.7	23	6.5	0.7	5.0	7.9
Cleithral Width (8–9)	26.4	23	28.5	1.2	25.6	30.7
Head-pectoral Length (1–12)	25.8	23	27.2	0.7	25.8	28.5
Thorax Length (12–13)	22.2	23	22.7	1.1	20.2	24.8
Pectoral-spine Length (12–29)	31.2	23	32.0	1.2	29.6	34.7
Abdominal Length (13–14)	22.0	23	20.3	0.6	19.2	22.0
Pelvic-spine Length (13–30)	27.9	23	27.2	1.2	25.3	29.0
Postanal Length (14–15)	39.0	23	35.2	1.5	32.5	39.0
Anal-fin spine Length (14–31)	18.1	23	16.3	1.1	14.8	19.1
Dorsal–pectoral Distance (10–12)	24.5	23	26.8	1.6	23.8	29.7
Dorsal spine Length (10–11)	32.6	22	32.7	1.7	28.8	35.7
Dorsal-pelvic Distance (10–13)	24.9	23	22.3	1.4	18.8	24.9
Dorsal-fin base Length (10–16)	29.3	23	28.9	0.9	27.0	30.9
Dorsal-adipose Distance (16–17)	15.3	23	15.2	1.1	12.0	17.4
Adipose-spine Length (17–18)	9.4	23	10.7	0.7	9.4	12.0
Adipose-upper caudal Distance (17–19)	16.2	23	17.1	1.0	15.3	19.3
Caudal-peduncle Depth (15–19)	10.8	23	11.7	0.5	10.8	13.2
Adipose-lower caudal Distance (15–17)	22.9	23	23.0	0.9	21.6	24.8
Adipose-anal Distance (14–17)	20.8	23	20.3	0.5	19.3	21.0
Dorsal-anal Distance (14–16)	14.9	23	15.0	0.6	14.2	16.3
Pelvic-dorsal Distance (13–16)	27.5	23	22.5	1.3	20.8	27.5
% Head Length						
Head-eye Length (5–7)	31.4	23	36.3	2.0	31.4	41.3
Orbit Diameter (4–5)	18.9	23	21.9	1.0	18.9	23.7
Snout Length (1–4)	53.7	23	56.3	1.5	53.7	59.5
Internares Width (2–3)	11.2	23	11.3	0.5	10.3	12.3
Interorbital Width (5–6)	43.0	23	46.8	1.8	43.0	49.6
Head Depth (7–12)	59.6	23	67.0	2.6	59.6	73.5
Mouth Length (1–24)	41.9	23	41.9	1.8	38.0	46.1
Mouth Width (21–22)	42.4	23	43.1	2.2	36.3	46.0
Barbel Length (22–23)	16.9	23	17.5	2.5	12.6	22.3
Dentary Tooth Cup Length (25–26)	11.3	23	11.1	1.5	7.9	13.8
Premaxillary Tooth Cup Length (27–28)	10.6	23	12.0	1.5	9.2	15.4

Eye moderately sized (orbit diameter 18.9 ± 1.0% of head length), dorsal rim of orbit forming tall crest that continues forward to area just anterior of nares as low, rounded ridge. Iris operculum present. Interorbital space with slight, rounded, median hump that is contiguous with ridge of parieto-supraoccipital. Parieto-supraoccipital pointed posteriorly with posterior point raised above nuchal region in small crest. Infraorbitals, frontal, nasal, compound pterotic and parieto-supraoccipital supporting odontodes. Preopercle generally supporting a single column of odontodes that generally decrease in number with increasing body size; largest specimen with some odontodes located posterodorsally on preopercle and two individuals without preopercular odontodes. Opercle with one to three rows of odontodes with numbers of rows and numbers of odontodes generally decreasing with increasing body size; largest individual without odontodes.

Lips covered with short, wide papillae. Lower lip wide, reaching just to or slightly short of pectoral girdle; upper lip narrow. Edge of lower lip smooth. Maxillary barbel only barbel present, reaching about two-thirds of distance to gill opening from base of barbel.

Median plates 24–26 (mode 24). Plates unkeeled, but first four or five plates of mid-ventral series bent to form slight ridge. Five caudal peduncle plate rows. Plates on all dorsolateral surfaces of body. Throat naked. Abdomen mostly naked except for a line one to three platelets wide along and slightly posterior to anterior margin of pectoral girdle, few uneven rows of platelets ventral to ventral plate series, patch of platelets below posterior section of pelvic girdle; number of platelets on abdomen increases with body size. Evertible cheek plates supporting hypertrophied odontodes evertible perpendicular to head. Cheek odontodes 17–40 (mode 33). Longest evertible cheek odontode almost reaching vertical through posterior edge of pectoral-fin spine. Hypertrophied cheek odontodes relatively weak. Odontodes slightly longer than average body odontodes present along dorsal-, adipose-, pelvic-, caudal-, and pectoral-fin spines; larger individuals with hypertrophied odontodes at tip of pectoral spine.

Dorsal fin ii,7; dorsal spinelet *V*-shaped, dorsal-fin locking mechanism present, last ray of dorsal fin not reaching or just reaching preadipose plate when adpressed. Adipose fin with single preadipose plate and moderately long spine. Caudal fin i,14,i; caudal fin forked, ventral lobe longer than dorsal lobe; dorsal and ventral procurrent caudal-fin rays five. Pectoral fin i,6; pectoral-fin spine reaching just posterior to pelvic fin when adpressed ventral to pelvic fin. Pelvic fin i,5; pelvic-fin spine extending one to two plates posterior to anal fin when adpressed. Anal fin i,4; unbranched anal-fin ray slightly shorter than first branched ray.

Teeth bicuspid with lateral lobe one-half to three-quarters length of medial lobe and lateral cusp half width of medial cusp. Eighteen to 32 left dentary teeth (mode 31; 1 of 23 with less than 25); 22–35 left premaxillary teeth (mode 32; 1 of 23 with less than 25); all specimens with at least one jaw ramus having 25 or more teeth.

**Color.** Base color reddish brown. Head and nape mottled dark brown with distinct, medial dark line along mesethmoid and slightly less distinct lines from lateral portion of naris to lip mark. Parieto-supraoccipital crest darker than surrounding areas. Lip with dark mark consisting of two or three ovoid dashes of brown, lateral portions continue as line to lateral portions of nares (Fig. 6). Dark portion of lips separated from mesethmoid line by distinctly lighter *C*-shaped region (Fig. 6), which may continue less distinctly between mesethmoid line and lines lateral to nares. Lips may also have other spots. Head colors less distinct in larger specimens. Body with four distinct, oblique bars, first below anterior of dorsal fin, second below posterior end of dorsal fin and anterior part of interdorsal space, third beginning at preadipose plate to about posterior edge of adipose spine, and fourth at end of caudal peduncle; first and second bars fade into a dark wash ventrally connecting the two bars; third and fourth bars continuing around caudal peduncle; bars connected at median plate series. Secondary bars sometimes present between any two primary bars, but generally not as dark, darker ventrally than dorsally; posterior secondary bars darker than anterior; secondary bars may connect across caudal peduncle. Pectoral-fin spine dark brown with alternating, similarly-sized dark and light spots, spots continuing as bands on fin; two to five dark bands on fin with number increasing with body size. Pelvic fin as pectoral but with two to four dark bands. Dorsal fin as pectoral but with dark bands distinctly wider than light bands, three or four dark bands. Anal and caudal fins as pectoral, but with light bands wider than dark bands (one to three dark bands in anal and three to five bands in caudal). Adipose spine with dorsal section of third dark bar covering base in all specimens, with some also having dark spot at tip of spine; in largest individual (holotype) basal and distal spots combine along posterior margin of spine, with anterior edge having light space between spots. Abdomen mostly light, but with four to six large, faint blotches laterally and occasionally with one or two median faint blotches.

**Figure 2.**
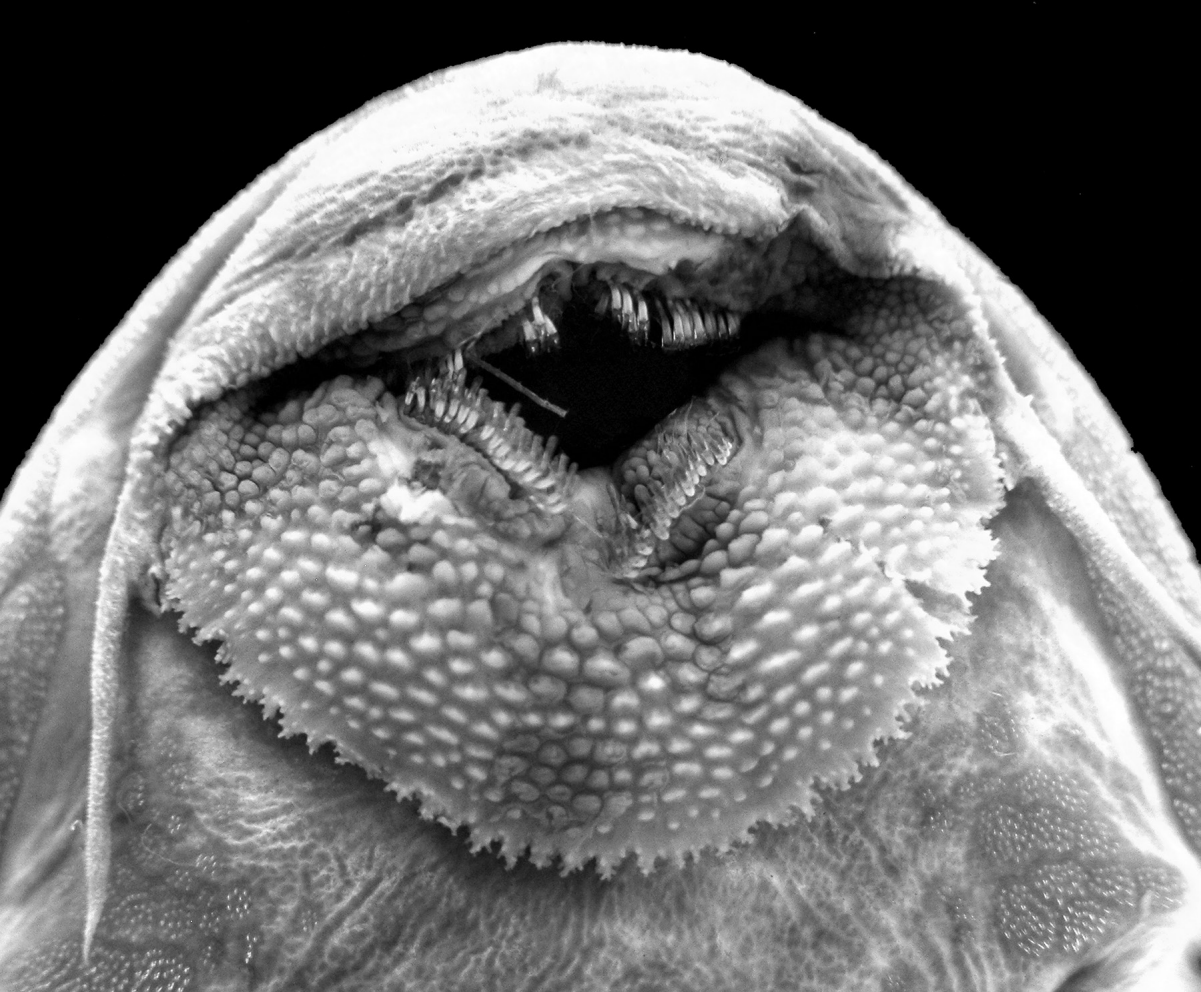
Mouth of *Peckoltia multispinis*, holotype, NMW 8952. Photo by M.H. Sabaj and K. Luckenbill.

**Figure 3.**
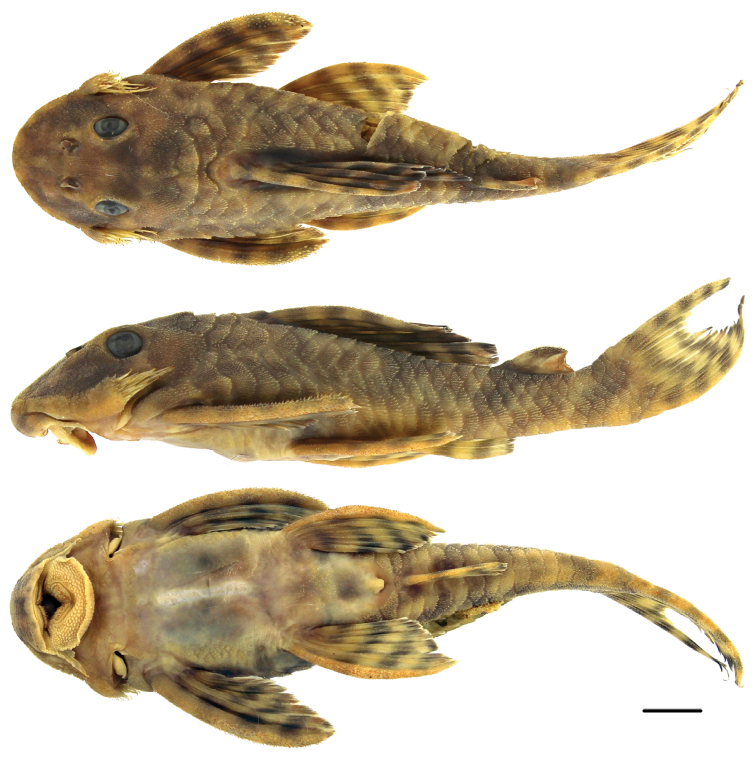
Holotype of *Peckoltia wernekei* sp. n., AUM 54314, 104.6 mm SL, dorsal, lateral, and ventral views. Scale = 1 cm. Photos by J.W. Armbruster.

**Figure 4.**
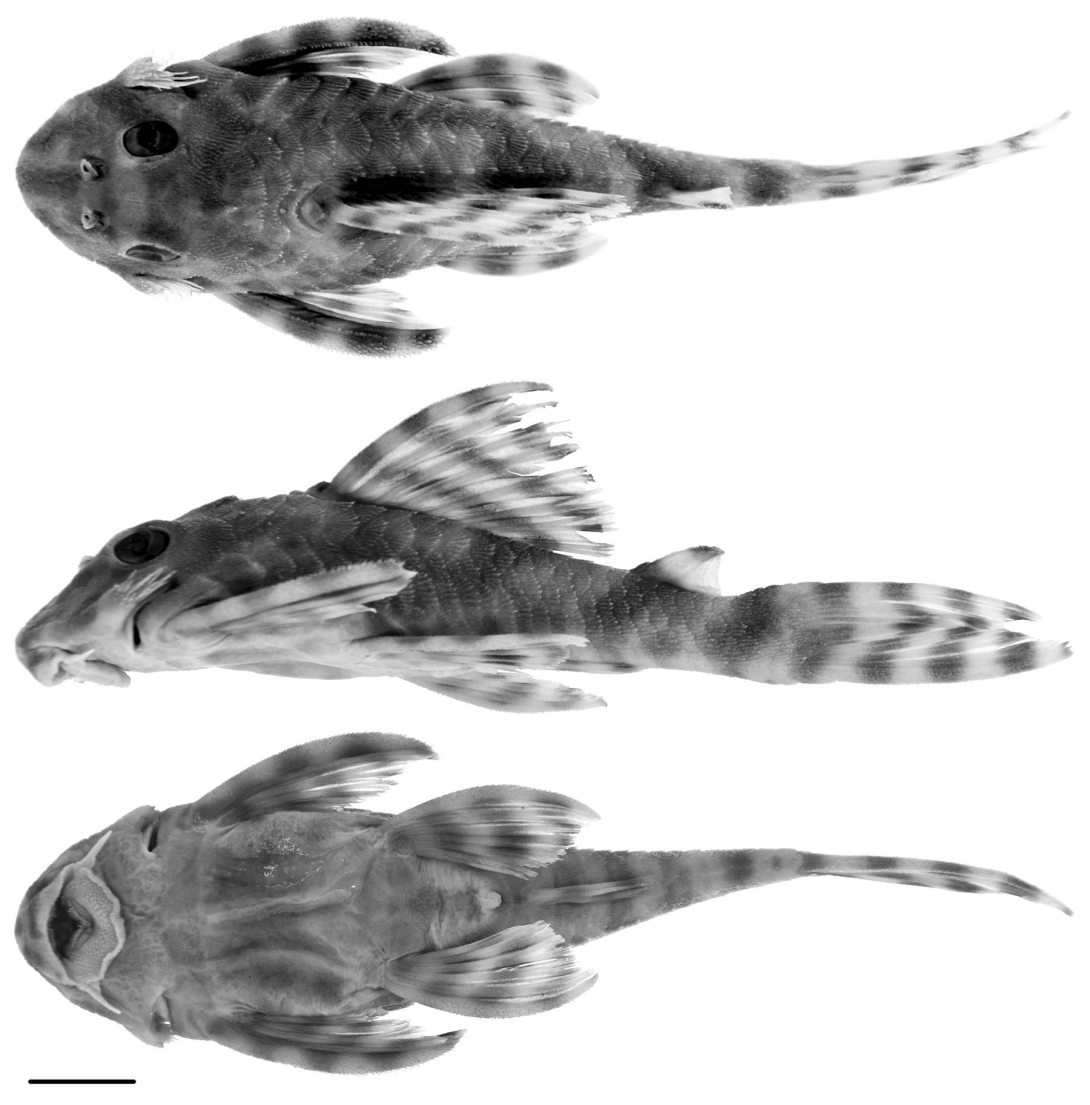
Paratype of *Peckoltia wernekei* sp. n., AUM 39313, 73.5 mm SL dorsal, lateral, and ventral views. Scale = 1 cm. Photos by J.W. Armbruster.

**Figure 5.**
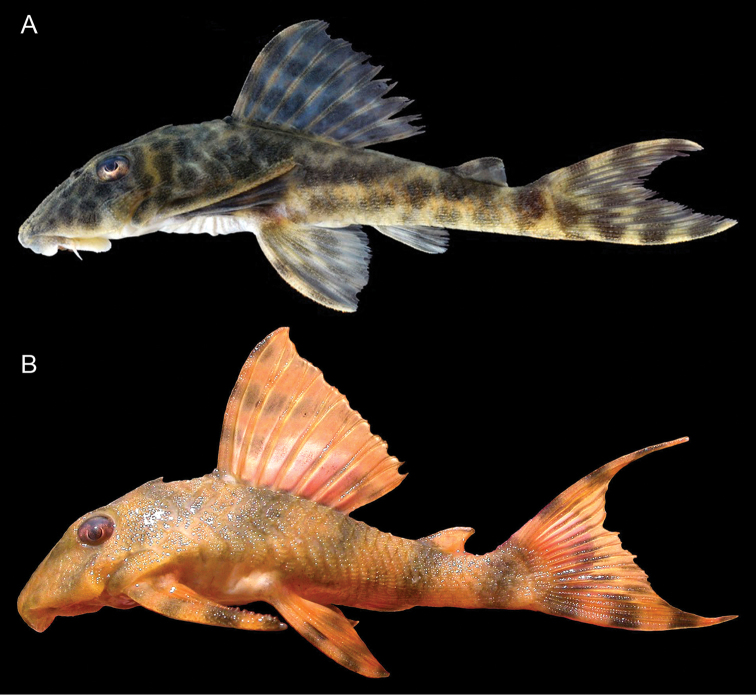
Live photos of **A** *Peckoltia lujani* (uncataloged), photograph by N.K. Lujan, and **B** *Peckoltia wernekei* sp. n. AUM 39313 (was used as a live photo of *Peckoltia vittata* in Armbruster 2008). Photograph by M.H. Sabaj Pérez.

**Figure 6.**
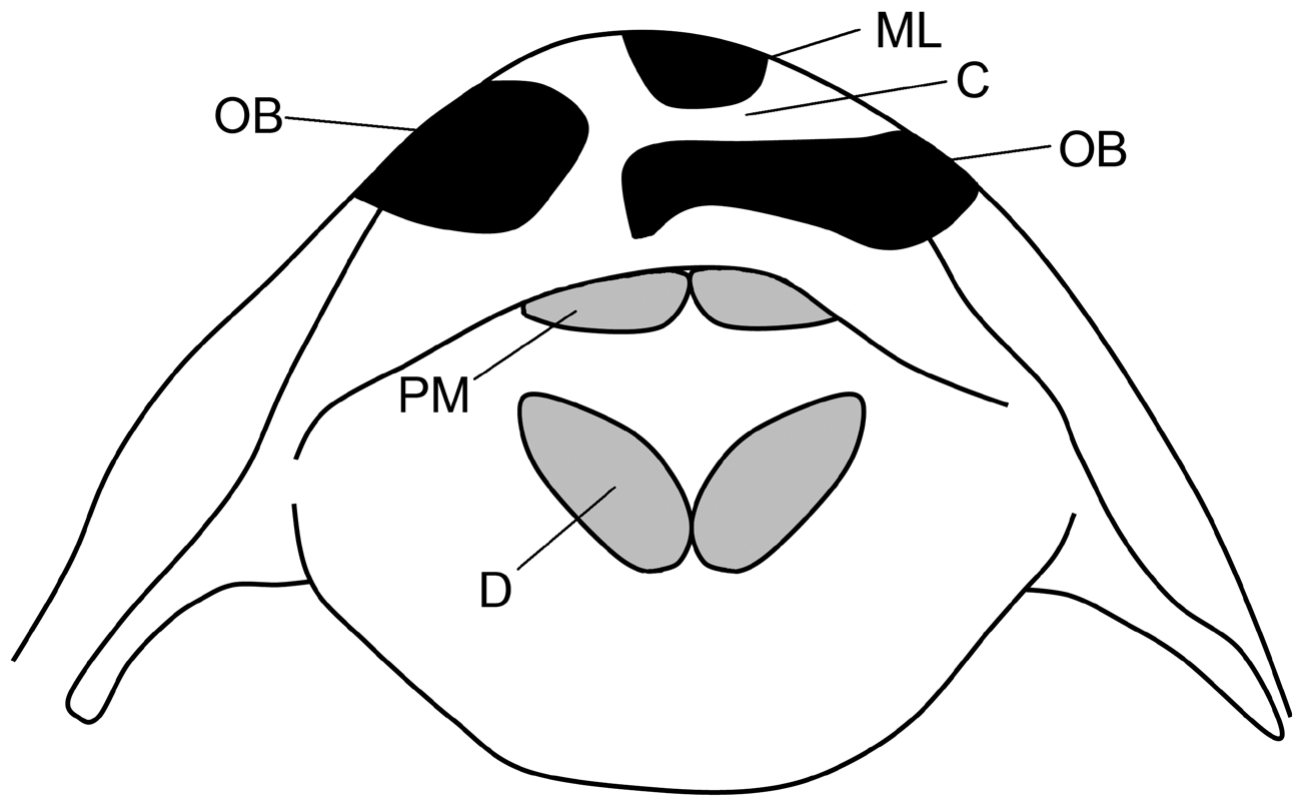
Schematic of the mouth of *Peckoltia wernekei* based on the specimen in Figure 3. Dentaries (D) form less than a 90° angle with one another whereas premaxillae (PM) are relatively straight. A broad, broken band is formed from ovate blotches (OB) on the upper lip, separated from a line that runs along the mesethmoid (ML) by a *C*-shaped light space (C).

#### Sexual dimorphism.

None observed.

#### Distribution.

**Figure 7.**
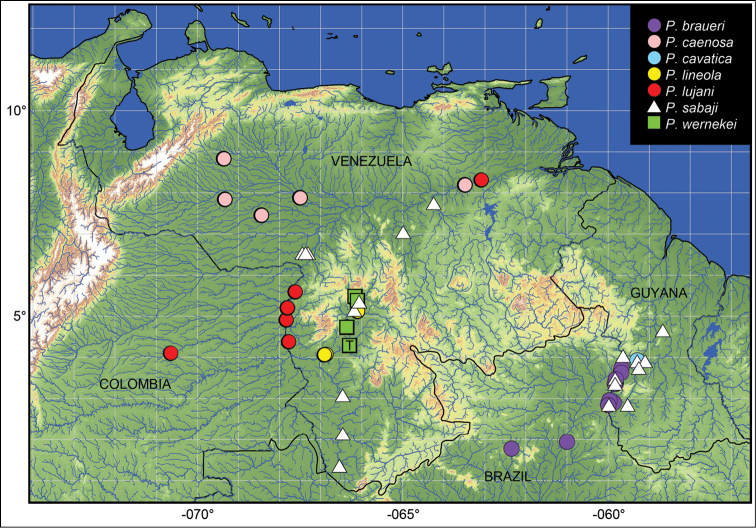
Distribution of *Peckoltia* around the western Guiana Shield. ‘T’ indicates type locality of *Peckoltia wernekei* sp. n.

Known only from the Ventuari River, a right-bank tributary of the upper Orinoco River in Amazonas State, Venezuela (Fig. 7).

#### Etymology.

Patronym honoring David C. Werneke, Collection Manager of Fishes at the Auburn University Museum, for his diligence, camaraderie and humor during three expeditions to the upper Orinoco Basin and for his long service as a Collection Manager at Auburn University.

### Key to the species of *Peckoltia* (after Armbruster (2008) and the present paper)

**Table:** 

1	Eyes reduced (orbit diameter <10% of HL); long caudal-fin filaments; lacking pigment or color reduced, light tan with very slightly darker saddles, mottled black and white or completely black	***Peckoltia pankimpuju***
–	Eyes normal (orbit diameter >13% of HL); caudal fin lacking filaments; color normal, with dark brown saddles, spots, or lines on a tan background)	**2**
2	Dentaries meeting at an angle less than 90° to just slightly greater	**3**
–	Dentaries meeting at an angle greater than 130°	**17**
3	Posterior margin of lower lip with finely branched fimbriate papillae (Fig. 2)	***Peckoltia multispinis***
–	Posterior margin of lower lip with simple fimbriae or smooth	**4**
4	Distinct round spots present on head and/or sides of body	**5**
–	Color pattern on head consisting of large blotches, saddles or lines, lacking distinct round spots on head or sides	**12**
5	Some spots on the parieto-supraoccipital and/or compound pterotic combining to form lines	**6**
–	All spots distinct, none combining to form lines	**7**
6	Lines on head most prominent on compound pterotic, not radiating from a central point on the parieto-supraoccipital; lines on head approximately same width or wider than pupil	***Peckoltia lineola***
–	Lines on the head most prominent on the parieto-supraoccipital where they radiate from a central point; lines on head narrower than pupil	***Peckoltia vermiculata***
7	Bands present on caudal fin (bands may be composed of spots arranged linearly)	**8**
–	Spots present on caudal fin separate, not forming bands	**10**
8	Lateral body surfaces with spots	***Peckoltia otali***
–	Lateral body surfaces with dorsal saddles	**9**
9	Spots on the dorsal fin; no spots on the abdomen; caudal fin lunate with upper caudal-fin spine longer than lower spine (usually the tail is broken and this character is not observable)	***Peckoltia furcata***
–	Bands on the dorsal fin; spots on abdomen of large juveniles and adults; caudal fin truncate or emarginated with lower caudal-fin spine longer than upper	***Peckoltia brevis***
10	Spots absent on head	***Peckoltia capitulata***
–	Spots present on head	**11**
11	Unworn teeth with lateral lobe about 2/3 or less the length of the medial lobe, longest cheek odontode not extending beyond cleithrum	***Peckoltia oligospila***
–	Unworn teeth with lobes approximately equal (lateral lobe just slightly shorter than medial lobe), longest cheek odontode extending beyond cleithrum	***Peckoltia simulata***
12	Caudal fin with dark bands much wider (approximately four or more times) than light bands; dorsal fin with white spots; abdomen with large dark spots with at least some spots merging to form vermiculations	***Peckoltia caenosa***
–	Caudal fin with dark and light bands of approximately equal width; dorsal fin with bands or uniformly colored; abdomen uniformly light or with faint dark spots that do not merge into vermiculations	**13**
13	Bold pattern of contrasting light and dark bars on body, usually five or more bars behind head, bars extending to or near ventrum without fading below the median plate series	***Peckoltia compta***
–	Dark saddles and lighter background colors not boldly contrasting, usually only four dorsal saddles behind head that fade below the median plate series and generally do not reach ventrum	**14**
14	Dark blotch between eyes and on snout, head mottled or with a bold patch of pigment in the form of an *E* on the snout with the central branch of the *E* located mid-dorsally and the top and bottom branches located just lateral to the nares, all three branches extending anteriorly and the main stem of the *E* running transversely centered on the nares; none of the plates of the head or nape outlined in black; dorsal and caudal fins without orange edge in life	**15**
–	Head and snout uniformly brown or with the plates outlined in black; *E* mark absent; posterior plates of the head and nape outlined in black; dorsal and caudal fins with an orange band at the edges in life	**16**
15	Upper lip mottled; teeth usually 24 or fewer per jaw ramus (only one specimen examined greater than 25); abdomen plated from throat to anus	***Peckoltia vittata***
–	Upper lip with a broken dark line of pigment, teeth usually 26 or more per jaw ramus (only one specimen examined with less than than 25); abdomen mostly unplated (a few plates below pectoral girdle, between pelvic fins, and along sides of abdomen)	***Peckoltia wernekei***
16	Vermiculations on compound pterotic; plates of head and nape not completely outlined in heavy black lines; caudal fin with at least one broken dark band; marginal orange bands of dorsal and caudal fins narrow	***Peckoltia braueri***
–	No markings on compound pterotic; all bones of head and nape outlined in faint black lines; caudal fin without dark bands; marginal orange bands of dorsal and caudal fins wide	***Peckoltia cavatica***
17	Cheek odontodes evertible to less than 45° from head, 0–10 hypertrophied cheek odontodes, cheek odontodes very short, not extending to cleithrum (no longer than 15× length of those on lateral plates)	***Peckoltia relictum***
–	Cheek odontodes evertible to greater than 80° from head, 20 or more evertible cheek odontodes, cheek odontodes very long, extending to at least middle of cleithrum (much greater than 15× length of those on lateral plates	**18**
18	No spots or bands in dorsal fin	***Peckoltia ephippiata***
–	Spots or bands present on dorsal fin	**19**
19	Abdomen fully plated, caudal fin with upper lobe longer than lower lobe in adults	***Peckoltia furcata***
–	Abdomen with large naked areas, particularly between pectoral girdle and anus. Caudal fin with lower lobe longer than upper lobe	**20**
20	Head, sides and fins with large, bold spots	***Peckoltia sabaji***
–	Head mottled or with faint spots, sides with dorsal saddles, fins with bands	**21**
21a	No spots on the posterolateral surface of head and nape; pectoral spine in relaxed position angled dorsally (pointing at insertion of dorsal fin); and pectoral-fin spine reaching two or more plates of the ventral series beyond the pelvic base when adpressed ventral to pelvic fin	***Peckoltia greedoi***
–	Distinct spots on the posterolateral surface of head and nape; pectoral spine in relaxed position angled only slightly dorsally (pointing maximally to dorsal insertion of caudal fin); and pectoral-fin spine reaching less than one plate of the ventral series beyond the pelvic base when adpressed ventral to pelvic fin	**22**
22	Spots larger than twice naris diameter, often indistinct, irregularly spaced and merging into irregular shapes, especially posterior of dorsal-fin origin, spots generally dark gray on a light gray base	***Peckoltia lujani***
–	Spots on snout naris sized or smaller, distinct and evenly spaced, growing to larger than orbit size and/or merging into oblique bars posterior of dorsal-fin origin, spots generally dark brown on a light brown base	***Peckoltia* sp. n.** Meta L147

## Discussion

The disparity between the morphological and molecular phylogenies of Armbruster (2004; 2008) and Lujan et al. (2015) and Lujan et al. (in review) are likely due to homoplasy and convergence in the morphological dataset, and nowhere is this more obvious than in the upper Orinoco clade containing *Peckoltia lujani*, *Peckoltia wernekei* and *Peckoltia* sp. n. Meta L147 (Fig. 1). *Peckoltia wernekei* was included in *Peckoltia* *sensu* Armbruster (2008) because of its stocky body and dentaries forming an angle of less than 90° (Fig. 6). The molecular phylogeny found *Peckoltia wernekei* to be sister to *Peckoltia lujani*, a species with dentary tooth rows forming almost a straight line and a more elongate body (Fig. 5a). Despite the differences in jaw angle, which likely has ecological and functional repercussions (Lujan and Armbruster 2012), *Peckoltia wernekei* and *Peckoltia lujani* differ from one another by only one of approximately 600 base pairs (bp; <0.2%) sequenced from the mitochondrial 16s gene and three of approximately 1150 bp (<0.3%) sequenced from the mitochondrial cytochrome *b* gene, whereas *Peckoltia wernekei* has 9.6% cyt *b* sequence divergence from *Peckoltia vittata* in the Xingu and 7.5% divergence from *Peckoltia vittata* in the Madeira (Lujan et al. in review).

Given the nested phylogenetic position of *Peckoltia wernekei* among two other species with relatively straight tooth rows (*Peckoltia lujani* and *Peckoltia* n.sp. Meta L147; Fig. 1), its highly-angled dentary tooth row angle is likely derived and an example of convergence upon the condition observed in many other *Peckoltia*. Given the limited sequence divergence between *Peckoltia wernekei* and *Peckoltia lujani*, this jaw evolution has either occurred very quickly or there has been very recent mitochondrial introgression. Mitochondrial introgression was observed between the sympatric *Panaqolus koko* and *Peckoltia otali* (Fisch Muller et al., 2012) where the two species had similar sequences for the mitochondrial COI gene suggesting close relationships, but sequences in the nuclear F-Reticulon4 gene showed no such close relationship. In this case, *Peckoltia wernekei* and *Peckoltia lujani* are not known to be sympatric and nuclear genes are also very similar (≤0.3% divergence in RAG1, RAG2 and MYH6; Lujan et al., in review), so we believe that the genetic similarity between these species is indicative of close evolutionary relationships and not hybridization. If such a pattern can be seen among closely related species, then many elements of jaw morphology are likely to be convergent across the phylogeny. Indeed, highly-angled jaws were seen in several clades even within the morphological phylogeny (Armbruster 2004; 2008). We are now testing this hypothesis with a phylogenetically explicit examination of jaw morphological diversity across the Hypostominae.

*Peckoltia wernekei* is illustrative of an increasingly clear biogeographical pattern in which the fish fauna of the Orinoco River upstream of its confluence with the Ventuari undergoes replacement or turnover between this confluence and the large Autures Rapids downstream. We hypothesize that this is due to an environmental filter similar to that documented by Winemiller et al. (2008) in the nearby Casiquiare Canal. The limnology of the Casiquiare Canal displays a gradient from relatively neutral or slightly acidic pH with few tannins at its origin in the upper Orinoco, to highly acidic pH with high tannin load at its confluence with the Negro River. Likewise, the physicochemical parameters of the clearwater upper Orinoco River shift a short distance downstream from the Ventuari River in response to its confluence with one major blackwater tributary (the Atabapo River) and one major whitewater river (the Guaviare/Inirida River) along its left-bank.

Many loricariid species are known only from the Ventuari River and nearby reaches of the Orinoco main channel. These include: *Baryancistrus demantoides*, *Baryancistrus beggini*, *Hemiancistrus subviridis*, *Hypancistrus contradens*, *Hypancistrus furunculus*, *Hypancistrus lunaorum*, Leporacanthicus cf. galaxias, *Leporacanthicus triactis*, *Hypancistrus vandragti*, *Peckoltia lineola*, *Pseudolithoxus tigris*, and *Pseudancistrus pectegenitor* (Armbruster 2008, Armbruster et al. 2007, Lujan and Armbruster 2011, Lujan and Birindelli 2011, Lujan et al. 2007, 2009, Werneke et al. 2005). At least two other species have a disjunct distribution inclusive of the Ventuari River and the Caura River but are absent from intervening reaches of the Orinoco River main channel (*Limatulichthys nasarcus*, and *Pseudolithoxus anthrax*; Lujan and Birindelli 2011, Londoño-Burbano et al. 2014), and in four of these instances there is strong morphological or molecular evidence that sister species are allopatrically distributed upstream vs. downstream of the confluence of the Orinoco and Atabapo/Guaviare/Inirida rivers. In addition to the *Peckoltia lujani*/*Peckoltia wernekei* pair, there is *Hypancistrus debilittera* and *Hypancistrus furunculus*, *Pseudolithoxus kelsorum* and *Pseudolithoxus tigris*, and *Hemiancistrus* sp. n. L128 (Dignall 2014) and *Hemiancistrus subviridis*.

## Supplementary Material

XML Treatment for Peckoltia wernekei

## Supplementary materials

Supplementary material 1 Morphometrics and meristics for species of *Peckoltia* and *Etsaputu* Data type: (measurement/occurence/multimedia/etc.) Explanation note: Morphometirc and meristic data based on Armbruster (2003) for all specimens of *Peckoltia* examined. This dataset is made available under the Open Database License (http://opendatacommons.org/licenses/odbl/1.0/). The Open Database License (ODbL) is a license agreement intended to allow users to freely share, modify, and use this Dataset while maintaining this same freedom for others, provided that the original source and author(s) are credited.Jonathan W. Armbruster, Nathan K. Lujan

Supplementary material 2 Locality information for all collections of the three species described in this paper Data type: Occurences Explanation note: Database containing catalog numbers, numbers of specimens, and localities for *Peckoltia wernekei*. This dataset is made available under the Open Database License (http://opendatacommons.org/licenses/odbl/1.0/). The Open Database License (ODbL) is a license agreement intended to allow users to freely share, modify, and use this Dataset while maintaining this same freedom for others, provided that the original source and author(s) are credited.Jonathan W. Armbruster, Nathan K. Lujan
